# When Three Isn’t a Crowd: A Digyny Concept for Treatment-Resistant, Near-Triploid Human Cancers

**DOI:** 10.3390/genes10070551

**Published:** 2019-07-19

**Authors:** Kristine Salmina, Bogdan I. Gerashchenko, Michael Hausmann, Ninel M. Vainshelbaum, Pawel Zayakin, Juris Erenpreiss, Talivaldis Freivalds, Mark S. Cragg, Jekaterina Erenpreisa

**Affiliations:** 1 Latvian Biomedical Research and Study Centre, LV-1067 Riga, Latvia; 2 R.E. Kavetsky Institute of Experimental Pathology, Oncology and Radiobiology, National Academy of Sciences of Ukraine, 03022 Kyiv, Ukraine; 3 Kirchhoff Institute for Physics, Heidelberg University, D-69120 Heidelberg, Germany; 4 Institute of Cardiology and Regenerative Medicine, University of Latvia, LV-1004 Riga, Latvia; 5 Riga Stradins University, LV-1007 Riga, Latvia; 6 Clinic IVF-Riga, LV-1010 Riga, Latvia; 7 Centre for Cancer Immunology, University of Southampton, Southampton SO16 6YD, UK

**Keywords:** near-triploid cancer, radioresistance, chemoresistance, reprogramming, digyny, polynuclear cancer cells, tripolar mitosis, pedogamy, tumor blastomeres

## Abstract

Near-triploid human tumors are frequently resistant to radio/chemotherapy through mechanisms that are unclear. We recently reported a tight association of male tumor triploidy with XXY karyotypes based on a meta-analysis of 15 tumor cohorts extracted from the Mitelman database. Here we provide a conceptual framework of the digyny-like origin of this karyotype based on the germline features of malignant tumors and adaptive capacity of digyny, which supports survival in adverse conditions. Studying how the recombinatorial reproduction via diploidy can be executed in primary cancer samples and HeLa cells after DNA damage, we report the first evidence that diploid and triploid cell sub-populations constitutively coexist and inter-change genomes via endoreduplicated polyploid cells generated through genotoxic challenge. We show that irradiated triploid HeLa cells can enter tripolar mitosis producing three diploid sub-subnuclei by segregation and pairwise fusions of whole genomes. Considering the upregulation of meiotic genes in tumors, we propose that the reconstructed diploid sub-cells can initiate pseudo-meiosis producing two “gametes” (diploid “maternal” and haploid “paternal”) followed by digynic-like reconstitution of a triploid stemline that returns to mitotic cycling. This process ensures tumor survival and growth by (1) DNA repair and genetic variation, (2) protection against recessive lethal mutations using the third genome.

“*All basic traits inherent in cancer cells are displayed in gametes and vice versa*”

Janis-Olgerts Erenpreiss [1]

## 1. Introduction

**Figure 1:**
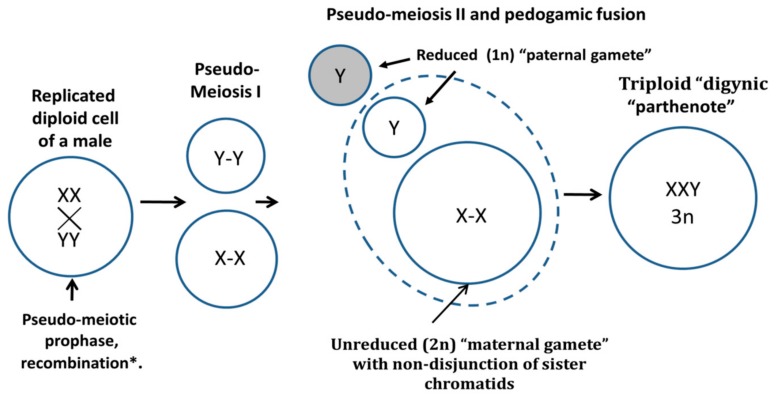
A conceptual schematic of the digyny-like formation of XXY triploid karyotypes in somatic male tumors revealed in the Mitelman karyotypes database data [10,11]. The reprogrammed male tumour cell triggers from G2-phase the aberrant molecular pathway of meiosis (here termed pseudo-meiosis), undergoes recombination between cohesed sisters and homologues *, pseudo-meiosis I segregating maternal, and paternal progenies with cohesed sister chromatids, reduction to haploidy of the “paternal gamete” in the pseudo-meiosis II and its pedogamic fusion with the unreduced diploid “maternal gamete” resulting in triploid “digynic parthenote”. * For recombination details, which are aberrant, see [12].

Organismal triploidy in humans is known to be lethal and causes early spontaneous abortions [2]. In contrast, aneuploidy is a well-tolerated characteristic hallmark of most human tumors [3,4,5]. Moreover, many established tumor cell lines used as models for cancer research or pharmacological studies exhibit near-triploidy [6] and many chemotherapy-resistant cancers display it in vivo [7,8,9]. In the accompanying article [10] we recently presented data for the origin of tumor triploidy based on the in silico meta-analysis of 2928 karyotypes from 15 malignant solid tumor types of male patients from the Mitelman database [11]. We provided evidence that triploidy very likely initially occurs through whole genome rearrangements when one paternal genome is added to two copies of the maternal genome (XXY karyotype correlating with near-triploidy, *r* = 0.88, *p* < 0.001). Such a karyotype can be formed by a digyny-like process (Figure 1). For female tumors, this karyotype should be triploid XXX (~69XXX). To obtain this outcome, a separation of parental genomes and sister chromatid non-disjunction in maternal genomes using an aberrant meiotic pathway can be presumed to occur at some stage of tumor development that involves the gametogenic reprogramming of somatic tumor cells. 

Below we briefly review the literature data which may give a hint for tackling the problem of cancer triploidy from this point. For a better understanding of the conceptual terms, we provide the reader with a Glossary.

### 1.1. Glossary

*Near-triploidy*—DNA content of the stem line, determined in relation to G1 diploidy DNA content, which is close to 1.5. [9,13]. For human karyotypes, near-triploidy is defined from the modal chromosome number, close to 3n = 69 chromosomes [10]. The confidence interval is chosen depending on the research object, method precision, and tasks.

*Digyny*—refers to the process of an unreduced maternal gamete becoming fertilized by a haploid sperm (reduced paternal gamete). The result of digyny is a triploid zygote.

*Unreduced maternal gamete*—the oocyte which does not undergo the second meiotic division and remains diploid.

*Digyny-like*—the process similar to digyny supposedly occurring in tumor cells of somatic origin reprogrammed to the epigenetic state of the germline (“maternal and paternal gametes”). 

*Parthenote*—an organism produced from an unfertilized ovum, which in human is incapable of developing beyond the early embryonic stages.

*Germline*—germ cells each descended or developed from earlier cells in the series, regarded as continuing through successive generations of an organism (life-cycles).

*Endoreduplication* (also referred to as endocycling) is the replication of the nuclear genome in the absence of mitosis, which leads to elevated nuclear gene content and polyploidy.

*Pseudo-meiosis* (somatic meiosis) is an asexual ploidy cycle. 

*Ploidy cycle*—includes the doubling(s) of ploidy (asexual by endoreduplication or sexual by fertilization) and its halving in reduction division(s). “The ploidy cycle provides the potential for orderly reduction which needs (1) cohesion of sister chromatids; (2) omission of DNA replication; and (3) pairing and recombination of homologous chromosomes, which is usually present but may be optional for somatic reduction” [14]. 

*Meiomitosis*—currently a poorly defined term used in particular for mitotic tumor cells, which undergo endoreduplication and in which the meiotic machinery is partially expressed. In these cells, chiasma occurs and sister chromatids are fully or partially linked together with cohesion. These cells reduce ploidy and again return to mitosis [15,16].

*Parasexual process*—any form of reproduction in which the recombination of genes occurs by a process other than the gamete fusion [17].

*Pedogamy*—reproduction by the fusion of gametes derived from the same parent cell [18]. 

*Triploid bridge*—an epistatic synonym of digyny. Unreduced egg cell formation in diploids represents the first step (a bridge) toward asexual reproduction [19]. It appears that a triploid bridge between sexual diploidy and asexual polyploidy can evolutionarily operate in both directions [20].

*Neosis*—the term introduced by R. Rajaraman [21,22] for a parasexual process in tumor cells resuming immortality and proliferation potential by particular de-polyploidization cycles of polyploidized senescing mother cells.

*Diplochromosome*—a chromosome containing four chromatids produced by two rounds of replication without chromatid separation (due to their unresolved cohesion).

### 1.2. Digyny in Developmental Biology and Human Diploid-Triploid Mosaicism

In developmental biology, the XXY triploid karyotype is referred to as “digyny”, which is the most common form of whole genome aneuploidy in plants and animals [20,23,24], however, it is rarely encountered in human pathology [25]. Digyny most often arises from aberrant fertilization of an unreduced diploid egg cell by a monoploid sperm leading to a sterile triploid organism [26]. Two rounds of endoreplication before meiosis instead of one also often favor failed reduction of the gamete in the unisexual reproduction [27]. In rare pathology of diploid-triploid human mosaics, a delayed digyny was also described—by incorporation of a pronucleus from a second polar body into one embryonic blastomere developed from an unreduced gamete [28].

In nature, a “triploid bridge” to asexual reproduction appears as an adaptation to adverse environmental conditions or can be artificially induced by them. In such cases, sexual organisms produce an increased number of unreduced oocytes so that after fertilization triploid sterile individuals appear [29]. Development “with a purpose” of the three-genome organisms seems strange because meiosis ensuring a reproductive process by recombination and ordered reduction “takes only two to tango” [30], while the third is odd. However, triploid individuals have two advantages—(1) the third genome is compatible with mitosis and diminishes the frequency of recessive lethal mutations and so favors clonal survival in a poor environment, (2) a sterile triploid conserves the energy otherwise needed for sex [20,24]. Therefore, the short-term advantage of digyny is even exploited in fish aquaculture when the female fish are treated with hydrostatic pressure, cold- or heat-shock to prevent their oocyte maturation in order to produce artificial triploid food-fish after fertilization [31].

From this data, the constitutive karyotype of near-triploid human male tumors ~69XXY found by us in silico in male malignant tumors and known chemoresistance of cancer triploidy motivated us to hypothesize that a process similar to digyny can occur in somatic tumor cells. Digyny is associated with gametogenesis. How can it be related to cancer?

### 1.3. Cancer Reprogramming To the Embryonality

Cancer (stem) cells can be reprogrammed to the epigenetic state of the cleavage embryo or even the germ cell [32]. This trait heralded by expression of totipotency cycle gene *POU5F1* [33], is started with the emergence of illicit tetraploidy triggered from G2-phase/mitotic slippage, particularly enhanced by genotoxic stress [34,35,36]. The reprogramming to the embryonal stemness of tumor cells was found in aggressive tumors in vivo [37] and recently documented by single-cell transcriptome sequencing in chemoresistant basal breast cancer and melanoma [38,39]. These facts correspond to the embryological theory of cancer and its gametogenic variant, known since the 19th century [1,40,41,42,43,44] and coming into power again in the 21st century [35,36,45,46,47]. Cancer cells were hypothesized to undergo a life-cycle-like process of reversible polyploidy for self-renewing “neosis” [21,22], producing a “germ” [35,48] comparable with sporogenesis [49,50]. In the following, this process will be termed pseudo-meiosis as displaying common features with meiosis. Pseudo-meiosis of somatic tumor cells is likely part of this asexual life-cycle as the relevant processes including cohesion of sister chromatids, recombination, and reduction divisions omitting the S-phase, with an expression of relevant meiotic genes, were reported for multiple treatment-resistant tumor cell lines [12,51,52,53,54], also in vivo [55,56]. Still, the details of the whole process (currently also termed meiomitosis) remain obscure [15,16].

### 1.4. Segregation of Haploid Genomes Is Coupled to Endoreduplication by Spindle Dysfunction

To get from diploidy to the digyny-like triploidy, segregation of haploid genomes should occur. Normally, it takes place in sexual meiosis but has been also described in the asexual life cycles, with meiotic elements. Segregation of haploid genomes by cycling polyploidy in the life cycle of radiolarian *Aulacantha* was first described by C. Grell [57]. This multi-step process was shown by him and further by others to operate with bi-chromatid chromosomes linked end-to-end in haploid genome entities, undergoing polyploidization (through a dysfunctional spindle), somatic pairing, followed by multipolar and bipolar mitoses, and final release of haploid spores [57,58]. Separation of autonomous duplicated parental haploid sets in diploid tissues induced by colchicine (which causes spindle dysfunction) or spontaneously by stress is known for plants, fungi, animals, [59], senescing cells, and was related by Walen [60,61] to human cancer. We recently provided evidence from human diploid embryonal carcinoma that segregation of parental genomes composed of bi-chromatid chromosomes with closed telomere ends, linked end-to-end, in a peculiar pro-metaphase possessing a dysfunctional mitotic spindle and forming a tetraploid cell, is followed by parental genome fusion and conjugation of homologues, recombination, and reduction divisions that restore diploidy [12]. From this data, it follows that separation of haploid genomes is likely evolutionary pre-programmed from the asexual life-cycles of haploid protists. It is coupled to endoreduplication, the cohesion of sister chromatids, and needs a dysfunctional spindle, which otherwise would separate them.

### 1.5. The Spindle Checkpoint Is Weak in the Preimplantation Embryo and Polyploidizing Tumor Cells

It is now established that a spindle arrest checkpoint is not fully functioning during the first four embryonal cleavage stages. The early blastomeres are genetically unstable [62,63,64] and frequently mixoploidy in vitro [65]. Moreover, in *Drosophila* the whole embryo remains multinucleated until de-polyploidization by cellularization, which occurs only at the blastocyst stage [66].

Importantly, during the first four cleavage divisions, along with the weakness of the spindle checkpoint, the parental genomes in normal mouse and human embryo also keep in mitotic plates as separate groups [67], that is while the blastomeres remain totipotent. Interestingly, the polyploidization of human tumor cells induced by the genotoxic challenge also usually proceeds until “a totipotency checkpoint”, the ploidy number (four endocycles) equivalent of the 32-cell morula, from which de-polyploidization including cellularization producing resistant mitotic progeny starts from day ~6–7 [68,69,70,71]. The multinucleated giant single cells matured to this point are capable of initiating tumor growth upon transplantation in animals [50,72,73].

### 1.6. Digynic Zygotes in Human In Vitro Fertilization (IVF) Clinic

Interesting information for our analysis of cancer triploidy is coming from the IVF clinic. Triploid zygotes are often observed there (with a frequency of 12%) and the most frequent cause is digyny—fertilization of unreduced oocytes by a haploid sperm [74]. Digynic triploid zygotes most often divide by tripolar mitosis [75].

In summary, the literature analysis suggests that endoreduplication coupled with reprogramming to embryonic germline totipotency and separation of parental haploid genomes through the dysfunctional spindle, and likely the tripolar mitosis, with poorly explored relationship to pseudo-meiosis, may be involved in the origin of digyny-like whole genome triploidy in radio-chemoresistant human tumors.

## 2. Materials and Methods

### 2.1. Patient Samples, Cell Lines, and Treatment

Breast cancer patient tissue specimens were collected after the patient’s informed consent was obtained in accordance with the regulations of the Committee of Medical Ethics of Latvia [9]. HeLa cervix carcinoma cells and Namalwa Burkitt’s lymphoma cells were obtained from ATCC. The HeLa cell culture was grown as a monolayer in flasks or on glass coverslips in F-10 medium (Hyclone Pittsburgh, PA, USA) containing 10% fetal bovine serum (FBS) (Hyclone, Pittsburgh, PA, USA) and antibiotics (Penicillin-streptomycin, Sigma P4333, Ronkonkoma, NY, USA) at 37 °C in a 5% CO_2_ humidified incubator (Sanyo, Watford, UK). ATCC cells were grown as suspension cultures in RPMI-1640 medium supplied with 10% FBS (Sigma).

For the experimental studies shown here, cells were maintained in the log phase of growth, and treated with a single, acute 10 Gy dose of gamma radiation using a Gulmay D3 225 X-ray source (Krefeld, Germany) at a dose rate of 0.77 Gy/min. After irradiation, cell cultures were maintained by replenishing culture medium every 2–3 days and sampled over a two-week period post-irradiation. For Aurora B-kinase detection in some specified experiments, the proteasome inhibitor 10μM lactacystin was added to the culture medium for 2 h before cell harvest.

### 2.2. Fluorescent In Situ Hybridization (FISH)

The fluorescent in situ hybridization (FISH) protocol has been described in detail previously [76]. In brief, HeLa cells were harvested, treated with 75 mM KCl at room temperature for 10 min and fixed with five changes of methanol/glacial acetic acid (3:1). The cell suspension was dropped onto slides and allowed to dry. Fluorescence labelled pericentric satellite DNA probes (Q-BIOgene–Molecular Cytogenetics/Diagnostics, Illkirch Graffenstaden, France) specific for chromosomes #6, #10, and #X were used and hybridized according to the manufacturer’s protocol. The cell nuclei were counterstained with DAPI (4′,6-diamidino-2-phenylindole) (ThermoFisher, Waltham, MS, USA) and embedded in the antifade mounting medium. The cover glass on the slides was sealed with rubber cement. These chromosomes were chosen as containing three normal copies and not participating in clonal markers [77]. The number of fluorescent chromosome labels per individual nuclei was counted in 500–800 cells 24 and 48 h after irradiation and compared to the non-irradiated control.

### 2.3. DNA Image Cytometry

Cells were grown on coverslips, prepared as cytospins or as smears and imprints on poly-L-lysine-coated microscopy slides (from breast cancer material), air-dried and fixed in ethanol: Acetone (1:1) for >30 min at 4 °C and then air-dried again. Slides were then hydrolyzed with 5N HCl for 20 min at room temperature. They were further washed in distilled water (5 × 1 min) and stained for 10 min with 0.05% toluidine blue (TB) in 50% citrate-phosphate McIlvain buffer at pH 4. After staining, samples were shortly rinsed in distilled water followed by blotting to dry and dehydration in butanol for 2 × 3 min at 37 °C. Samples were then incubated twice in xylene for 3 min at room temperature before being embedded in DPX. Digital images were collected at 100 × 10 magnification using L03-10 microscope (Ergolux, Leitz, Germany) equipped with DXC 390P color video camera (Sony, Tokyo, Japan) calibrated in the green channel. DNA content was measured as the integral optical density (IOD), using Image-Pro Plus 4.1 software (Media Cybernetics, Rockville, MD, USA). The stoichiometry of DNA staining was verified using the values obtained for metaphases compared to anaphases and telophases (ratio 2:0); arbitrary diploid (2C) DNA values were averaged from measuring 50 anaphases in non-treated tumor cells. The reference 2C DNA value and variation coefficient for DNA staining method were assessed in normal human leukocytes (≈2%). The device error was estimated at 0.5%. The integral error of the DNA staining for DNA index (DI) determination in breast cancer was assumed 10%; for HeLa mitotic cells the variation of the DNA C-value reached 20%.

### 2.4. Immunofluorescence

Immunofluorescence staining was performed as described [78]. The Aurora B kinase antibody (ab2254) was purchased from Abcam (Cambridge, UK), α-Tubulin (T5168) from Sigma, and Lamin B1 (sc-6216) from Santa Cruz (Dallas, TX, USA). For microscopic observations, a fluorescence light microscope Ergolux L03-10 (Leitz) equipped with a color video camera (Sony DXC 390P, Tokyo, Japan) was used (Objective ×100, magnification ×1000). To capture fluorescent images, in addition to separate optical filters, a three-band BRG (blue, red, green) optical filter (Leica, Wetzlar, Germany) was used.

### 2.5. In Silico Study of the Mitelman Database

The in-silico study of the Mitelman database was performed as described in the accompanying article [10,11]. *Nota bene*: For the sex chromosomes, chromothripsis was specifically filtered out by our bioinformatic pipeline, to allow us to document whole genome rearrangements [10].

## 3. Results

After assuming that a digyny-like process can convert diploid tumor cells into triploidy “digynic parthenotes”, we suggest as a working hypothesis, that on its side, the near-triploid tumor cell line uses the endoreduplication platform to create the diploid stemline, capable of undergoing the recombinatorial pseudo-meiosis and reciprocal exchange with “digyny”. 

We report below some data relevant to this hypothesis from our studies of male tumors from the Mitelman database and from chemoresistance study of breast cancer tumors in patients. Next, we searched for the evidence and mechanisms of the reversible conversion of triploidy into diploidy in the well-known tumor model cervical carcinoma HeLa. We provoked resistant near-triploid cervical carcinoma HeLa cells (with a modal chromosome number 69 [79]) by one hit of ionizing irradiation (10 Gy) to follow the cell division events over the two-week course.

### 3.1. A Triploid Cell-Line May Coexist with Cycling Diploidy in Patient Tumors

#### 3.1.1. Male Tumors (Renal Carcinoma)

Analysis of histograms of modal chromosome numbers in large 15 cohorts of monoclonal tumor types from the Mitelman database presented in [10] revealed near-diploid, near-triploid, and also near-tetraploid karyotypes in each tumor type. One can suggest that “monoclonal” near-triploid tumors could contain or give rise to a small amount of diploid and tetraploid sub-clones (which may be hidden in a dormant state). Some examples of the reported polyclonal karyotypes from male renal carcinoma with near-triploid XXY, diploid, and tetraploid clones from the Mitelman karyotype database (exemplified in Table 1) are compatible with this assumption.

#### 3.1.2. Female Tumors (Breast Cancer)

The coexistence of the cycling triploid with the cycling diploid stemline was clearly seen in our study of breast cancer in patients [9]. In cases of chemoresistance, a cycling and endocycling triploidy (3C, 6C, 12C, 24C) was found in each DNA cytometry histogram, both in the diagnostic biopsy and material from the same patient post-neoadjuvant therapy (Figure S1). In one case (case 30), the triploid clone evolved from a very small cycling sub-clone in initial tumor biopsy to become the dominant stemline after non-successful chemotherapy (Figure S1). The study also revealed that in breast cancer tumors with a near-triploid stemline the hyper-tetraploid cell fraction (DNA C value > 4.5) was 4-times larger (*p* = 0.003) than in near-diploid tumors [9], indirectly pointing towards the role of endocycles in the origin of triploidy. Similarly, Kim et al. [39], in a single cell study, noticed the emergence of the triploid clone in the case of resistant to therapy basal breast cancer. 

### 3.2. Diploid, Tetraploid, and Haploid Cell Nuclei Are Induced after Irradiation of Near-Triploid Cervical Carcinoma HeLa Cells

**Figure 2:**
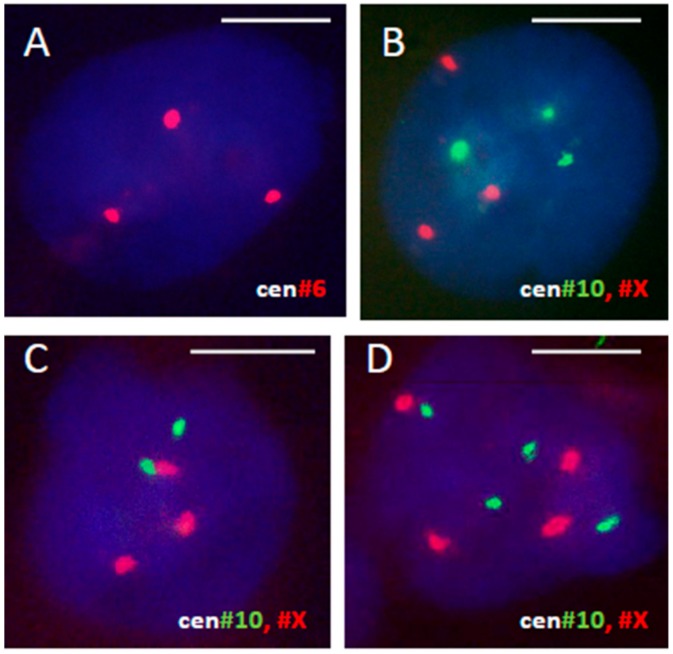
Representative fluorescent in situ hybridization (FISH) patterns revealing (**A**,**B**) triploid, (**C**) diploid, and (**D**) tetraploid cell nuclei 48 h after 10 Gy hit in HeLa cells using centromeric labels for three chromosomes, #6, 10, X.

The model of a single 10 Gy irradiation hit of HeLa cells has been previously established in the prolonged life-imaging digital studies in the lab of Ianzini and MacKay [80], while HeLa cell response in this model during two weeks post-damage using life-imaging, DNA cytometry, and immunofluorescence was further reported in the joint studies with Ianzini/MacKay, our and Hausmann labs in Heidelberg [78,81]. In particular, it was shown that HeLa cells polyploidize in response to this irradiation hit, while cell clonal regrowth starting from day 7–9 is provided by de-polyploidization of a small proportion of still-persisting giant cells. In the current research, following treatment of HeLa cells with a single dose of 10 Gy irradiation, cells were sampled and FISH was applied for centromeres of three chromosomes #6, #10, #X (presented as triples without markers in a HeLa carcinoma genome). The relative proportions were determined in 500–800 cell nuclei for each chromosome label per nucleus. The representative FISH patterns of triploid, diploid and tetraploid nuclei are shown on Figure 2. 

**Figure 3:**
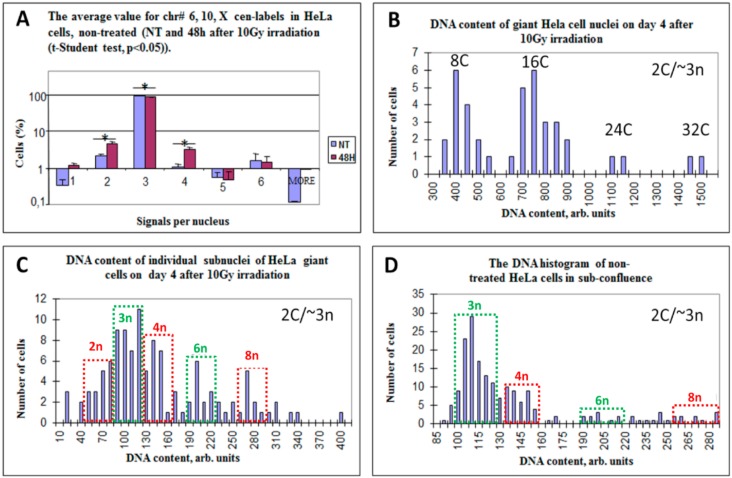
Endoreduplication and emergence of diploidy in the near-triploid HeLa cell line after 10 Gy-irradiation. (**A**) The decrease of the proportion of triploid nuclei and a corresponding increase of the proportion of diploid and tetraploid cell nuclei are presented as an average value for chr #6, #10, #X cen-labels in non-treated controls and 48 h after irradiation (*t*-student-test, *p* < 0.05). Percentage of cells is presented in logarithmic scale. (**B**) DNA content (presented as integrated optical density in arbitrary units) in giant cell nuclei on day four post-irradiation. The ploidy of 2C, roughly corresponding to ~3n, was determined from anaphase halves of bipolar mitoses of the control (*n* = 50). (**C**) Heterogeneity of the giant cell sub-nuclei on day four post-irradiation (obtained from 116 giant cells as determined by DNA cytometry, and expressed and converted for convenience into ~ploidy numbers showing triploid (3n) and hexaploid (6n) nuclei along with about 50% of diploid nuclei and their multiples (2n, 4n, 8n); (**D**) Non-treated HeLa cells harvested in sub-confluence, composed of the dominant 3n stemline, while the diploid stemline endocycling to 8n is also seen (mostly accumulated in the 4n-fraction). On (**C**,**D**), the diploid endocycling stemline fractions are enframed in red, while triploid, in green dashed boxes.

As seen in Figure 3A, the non-treated population consisted on average of 94% cell nuclei, trisomic by all three centromeric-labels (NB: hexa-somy indicating to triploidy cycling was also observed), however a very small proportion of diploid (disomic) and tetraploid (tetrasomic) cell nuclei and a negligible amount of haploidy (monosomy) were also present. At 48 h after 10 Gy irradiation the proportion of triploid cells decreased to 88% on average, while the proportion of diploid and tetraploid cells correspondingly increased. In addition, a tiny subpopulation of haploid nuclei also appeared (Figure 3A). When analyzed by χ-squared test (Table 2), the change of each label from non-treated culture to 48 h post-irradiation showed its statistical significance, while all three labels changed in accord. This leads us to suggest that triploid cells began the rearrangement of the whole genomes to produce diploid and tetraploid fractions.

We were therefore interested to see if and how this tendency developed and examined the DNA ploidy by in situ cytometry on further days after irradiation.

### 3.3. Multinucleated Giant Cells Contain DNA with Odd and Even Ploidy Numbers

**Figure 4:**
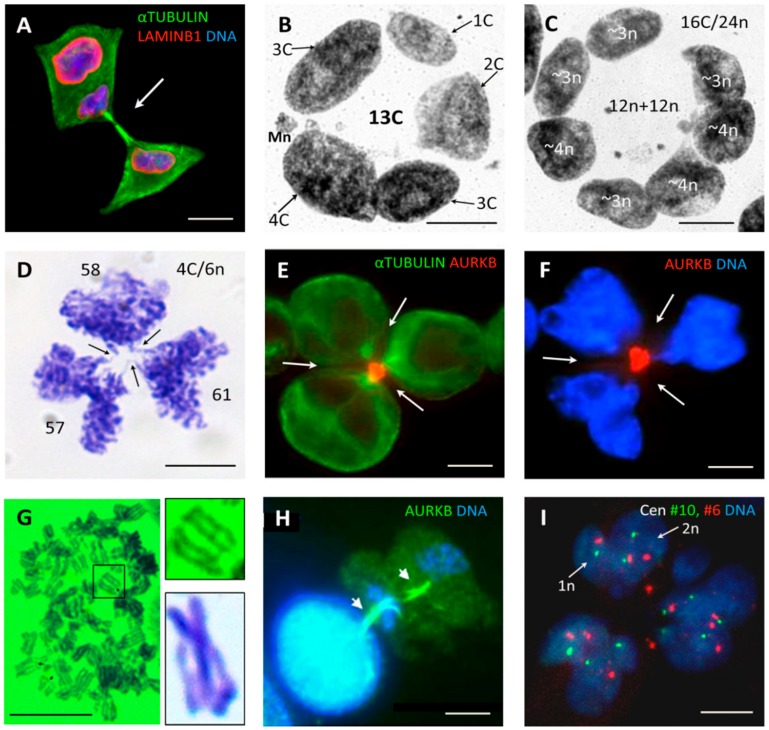
Divisions and multi-nucleation of the genotoxically damaged tumor cells. (**A**) HeLa cell, on day two post-irradiation (10 Gy): One subnucleus in a bi-nucleated cell is being divided (arrow). (**B**) A processed for image analysis (DNA bridges were deleted) multinucleated giant HeLa cell nucleus on day six post-irradiation containing sub-nuclei with even and odd proportions of DNA content, along which a near-haploid (~1C) sub-nucleus and a micronucleus (Mn) were seen; (**C**) A processed for image analysis (DNA bridges were deleted) multinucleated giant HeLa cell on day nine post-irradiation containing four ~3n and three ~4n sub-nuclei with a similar sum DNA content; (**D**) The anaphase of the multipolar mitosis of an irradiated HeLa cell with chromosome bridges between the karyokinesis products of three bilobed groups, a finding indicative of the pair-wise fusion of segregated neighboring genomes (stained with toluidine blue for DNA). The arrows show indentations in the bridges between these pairwise fused products of three karyotomies due to incomplete or ongoing cleavage by radial cytotomy; (**E**,**F**) Radial cleavage furrows bringing three Aurora KB-positive mid-bodies to the fused one of their spindle poles together, potentially segregating the progeny with pedogamically fused bilobed nuclei; (**G**) The diplochromosomic metaphase of a HeLa cell on day five post-irradiation (10 Gy) (stained with Toluidine blue for DNA), resembling the diakinetic stage of meiosis with often intertwisted or DNA-bridged (arrows) two pairs of cohesed sisters. On insert enlarged: (a) The enframed bridged diplochromosome and an example of the chiasma found between diplochromosomes of the irradiated Namalwa cell line; (**H**) Two subsequent divisions of the tetraploid sub-nucleus of a giant Namalwa cell to a haploid pair of nuclei on day five post-irradiation (10 Gy) are seen, displaying two persisting mid-bodies (shown by the arrows). Persistence of two subsequent mid-bodies and ~ haploidy in DNA content of the final anaphase figures suggest that the second division closely followed the first division, omitting the S-phase. This sample was collected after 2-h treatment with the proteasome inhibitor—lactacystin; The brightness in the DAPI channel was enhanced to highlight small nuclei of the second anaphase; (**I**) The FISH sample for centromeres #6 and #10 of HeLa cells on day four post-irradiation (10 Gy) shows (arrows) haploid (monosomic) and diploid (disomic) lobes of a sub-nucleus resulting after multipolar karyokinesis. Bars = 10 µm. Figure 4A–C,H are reproduced from [78], with permission of the publishers.

As reported previously [78], on the first day most irradiated Hela cells were delayed in the G2-arrest, from day two, 82% of live-imaged HeLa cells (*n* = 645) underwent the chromosome-bridged post-telophase bi-nucleation, becoming tetraploid and many started the asynchronous bipolar divisions of two sub-nuclei (exemplified in Figure 4A). These divisions were also a-cytokinetic, and thus, by day three, 70% cells became octoploid (composed typically of two 4C sub-nuclei), in the following days the ploidy and multi-nucleation increased. By day 4–5, the multinucleated polyploid giant cancer cells (PGCC) composed of ~50% of the population and contained largely 8C, 16C, 24C, and 32C DNA in total (Figure 3B). DNA cytometry of individual sub-nuclei of the 4-day PGCCs reveals in them odd and even n-value numbers. C-value was determined from anaphase halves of the DNA content in bipolar mitoses of untreated cultures. Therefore, it should be kept in mind that for near-triploid HeLa, a 2C value roughly corresponds to ~3n. Subsequently, the derived ploidy numbers of the sub-nuclei were 2n, 3n, 4n, 6n, and 8n (Figure 3C). The sub-nuclei of PGCC were mostly bridged, indicating their origin from a common mother. Live-imaging showed that only 3% of cells in 72 h underwent fusion of non-sister cells [78]. Interestingly, the DNA histogram of the non-treated population taken at sub-confluence on day six (Figure 3D), also showed the same odd and even fractions (except 2n), however, the ploidy doubling fractions 6n and 8n were minor. The sub-nuclei in PGCCs with odd and even genome numbers were often arranged in giant cells radially and also frequently contained a near-haploid subnucleus (rarely two-three of them), and a few micronuclei (Figure 4B,C). 

In summary, a genotoxic challenge apparently caused the entrance of HeLa cells into endomitotic a-cytokinetic cycles, starting from bi-nucleated on day two and reaching by day three majorly 8C/12n and by day 4, 16C/24n ploidy. This response was heralded by the emergence of the increasing number of diploid, tetraploid, and octoploid subnuclei coexisting with triploidy (and also haploidy) in the same PGCCs. How could they achieve that? The answer was likely found in tripolar mitosis. 

### 3.4. Tripolar Mitosis of Endoreduplicated Cells May Convert Triploidy into Diploidy

**Figure 5:**
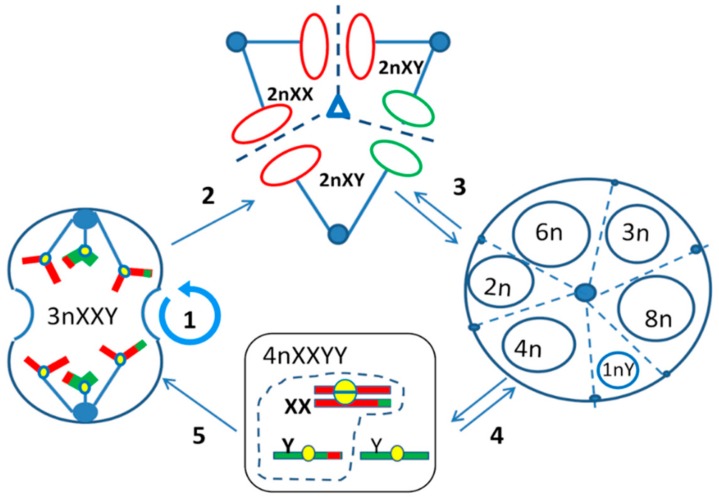
The Digyny Concept of meio-mitosis applied to near-triploid male tumor cells. (**1**) A digynic triploidy tumor cell line (3nXXY) undergoes mitotic cycles replicating and segregating sister chromatids for growth of the tumor mass. To perform recombinative renewal, it first closes telomere ends, and after replication segregates the genomes of the 3nXXY(x2) cell in (**2**) tripolar mitosis as 2:2:2 by pairwise fusing the haploid whole genome products of karyokinesis (red circles for arbitrary maternal, green, for paternal genomes) at each pole. (**3**) A diploid progeny (2nXY) endoreduplicates to 4n XXYY, and to 8n; a triploid subnucleus can also endoreplicate to 6n; 1n Y can be also (rarer) produced in tripolar mitosis (3:2:1); (**4**) 4n as a subnucleus of the polyploid giant cancer cell (PGCC) or as a free cell enters the recombinative pseudo-meiosis followed by one-step reduction division (for “maternal” sub-nucleus) and two-step reduction (for “paternal” sub-nucleus). A digyny-like fusion in 3n XXY subcell is processed (a dashed box). Alternatively (less likely), one non-recombined 1nY subnucleus from tripolar mitosis of a triploid cell (3:2:1) can fuse with two recombined, unreduced maternal genomes of a pseudo-meiotic diploid sub-cell. (**5**) A renewed digynic triploid cell restarts mitotic cycling, the process constitutive for tumor near-triploidy and favoring cell survival by compensating lethal recessive mutations. The near-diploid tumors can also enter this hybrid cycle induced through endoreduplication and multipolar mitosis, and thus be converted into triploid digynic stem-line if influenced by senescence, oncogenes, and genotoxic therapy.

Along with rare bipolar divisions, tripolar mitotic figures were seen in irradiated HeLa cells more often than in control, at the end of the second day and onwards, until recovery of the triploid clonogenic cell line. In some tripolar ana-telophase-like figures 48 h after irradiation, the chromosome bridges were observed between three bilobed chromosome groups, indicating the pairwise fusion of karyokinesis products (chromosome groups of a similar size) at each of three neighbor poles (Figure 4D). The arrowed indentations of the chromosome bridges between these pairwise fused products are due to incomplete, failed or ongoing cleavage by the radial cytokinesis. Cytometry revealed that three bilobed groups contained equal (with 4% variation) DNA content corresponding ~2n, testifying to the division of the replicated near-triploid cell into three diploid groups, where each likewise was formed by the fusion of two haploid genomes (6n:3 = 2n). The radial cleavage furrows potentially segregating the progeny with fused bilobed sub-nuclei and bringing three mid-bodies together are seen in (Figure 4E,F). Most tumor segregations appeared less precise, however, the reported observation shows namely that the tripolar mitosis of a replicated triploid 6n cell can convert triploidy into diploidy demonstrating a pair-wise fusion of haploid genomes (see schematic on Figure 5, central upper figure). If endoreduplication forms a larger 8C/12n cell, it can potentially produce by tripolar mitosis three 4n cells (12n:3 = 4n) or by tetrapolar mitosis four 3n cells (12n:4 = 3n). This may have occurred with the nucleus of a PGCC on Figure 4C containing four 3n and three 4n sub-nuclei, each group making the same 12n (~8C) DNA amount as measured by DNA cytometry. Those apparently represent the products of tripolar and tetrapolar divisions of two 8C subnuclei in the same giant cell. The prerequisites for such whole genome rearrangements would be the grouping and segregation of the haploid chromosome entities and non-separation of sister chromatids. Therefore, it is worth to mention as shown previously, that the chromosomal ends in acytokinetic multipolar mitoses of Hela cells were usually seen closed [12,81]. 

So, we found here that near-triploid HeLa cells begin to increasingly produce the diploid and tetraploid subfractions after irradiation, likely by a-cytokinetic tripolar mitosis of near-triploid cells endoreduplicated to 6n and 12n and that their proportion increased from ~ 6% on day two to ~40–50% on days 3–6. Thus, a tetraploid fraction composed of doubled parental genomes (~4nXXYY) could be reconstituted from a triploid one. It could further enter pseudo-meiosis and potentially produce a recombined unreduced diploid maternal pseudo-gamete and recombined reduced paternal haploid pseudo-gamete (this process is schematically represented for a male tumor on Figure 1). This process can take place either in the individualized cells—the diplochromosomic metaphases segregating bi-chromatids (presented on Figure 4G) may have a relationship to this process. However, it is not excluded that the whole pseudo-meiosis (recombination and reduction), as well as the digyny-like pedogamic process (fusion between an unreduced diploid “maternal gamete” and a haploid reduced “paternal gamete”), can take place at the site—on the platform of a polyploid giant cancer cell. A two-step asymmetric reduction division from a PGCC in irradiated lymphoma cells (reproduced here on Figure 4H) and occasional finding of two-parted HeLa giant cells sub-nuclei composed from a monosomic and disomic parts seen by centromere #6 and #10 FISH (Figure 4I) provide a hint for such an option. A non-sister fusion between diploid and haploid cells is not excluded as well, taking into consideration the very rare, but still found in vivo (Figure S2) divisions of free haploid tumor cells.

### 3.5. Summary of Results

The presented results provide the first evidence that in somatic human tumors the diploid and triploid compartments may constitutively co-exist and exchange the whole genomes in the same tumor. The study of irradiated near-triploid HeLa cells revealed that diploid subcells are produced after genotoxic insult in induced endopolyploid tumor cells through euploid whole genome segregations and fusions in tripolar mitosis.

## 4. Discussion

From the study of 15 male malignant tumor types with near-triploid XXY karyotypes assembled from the Mitelman karyotype database presented in the accompanying paper [10] and DNA cytometry of resistant triploid breast cancer, we suggested the association of the near-triploid XXY karyotypes (XXX for females) with a digyny-like pedogamic process—somewhat like an aberrant unisexual “fertilization”. From the literature analysis and experimental evidence presented here the whole process can be deduced as follows (Figure 5). The mitotically cycling near-triploid “digynic” tumor cell (Figure 5(1)), when challenged by stress, undergoes tripolar mitosis for the reconstruction of diploidy by segregating and fusing haploid genomes (Figure 5(2)). For that, these cells possibly use the platform of transient multinucleated giant cells enclosing resulting diploid and triploid sub-nuclei and their replicas (Figure 5(3)). In turn, the replicated diploid subnuclei/cells (4n) are capable of undergoing the recombinative pseudo-meiosis and creating pseudo-gametes. The parental pseudo-gametes (unreduced maternal and reduced paternal) pedogamically fuse, to reconstitute the triploidy digynic-like tumor stem line 3n XXY (Figure 5(4)). The latter, representing a triploid “parthenote”, stops further differentiation and returns in the genetically renewed, recombined form into a mitotic cycle (Figure 5(5)). On recovery, this mitotic clonogenic cycling becomes dominant again, increasing the tumor mass. It follows, that diploid and triploid sub-lines exchange whole haploid genomes. Somewhat in accord, Kroeger et al. [82] recently published the necessity for a hybrid epithelial-and mesenchymal state (Snail–Wnt-driven) for the growth of basal breast cancer suggesting the epithelial and mesenchymal cells cooperate as two sub-populations of tumors. Could they correspond to the diploid and triploid sub-lines? This is a question for future projects. Our hypothesis was stimulated by the previously published study of the chemoresistant triploid breast cancer [9] and the polyclonal karyotypes in male renal carcinoma, which were assessed here. Moreover, single-cell transcriptome sequencing tumor analysis also points to the polyclonal adaptive nature of cancer chemoresistance [83]. The current study on irradiated near-triploid HeLa cells brought us to PGCC—an analogue by its expression profile of the blastula embryo [35,36,47] and to tripolar mitoses in them.

Further, we should compare our results with a series of studies performed in the late 1960s to the mid-1970s [84,85,86,87], which essentially coincide with our observations. The authors used the multiple primary cultures of normal diploid mouse fibroblasts and Rhesus monkey kidney epithelial cells, applying DNA densitometry and cytogenetic analysis. They found that, contrary to bipolar mitosis occurring in diploid cells, the endoreduplicated tetraploid cells emerging in senescing cultures in a small proportion (~3% cells) from day 15–20 majorly undergo tripolar mitoses. Those were analyzed without applying spreading and spindle poisons. The euploid segregation of the genomes in haploid, diploid, and triploid ana-telophase groups by tripolar mitosis was reported in this article series published by two groups on two mammalian species. The genetic material of the initially diploid culture is most often segregating in tripolar mitosis as the multiples of haploid genomes in a 3n:3n:2n ratio—so, through endoreduplication the diploidy gives birth to triploidy. In turn, the resulting triploid cells most often segregate the genomes (after re-replication) by tripolar mitosis in a 2n:2n:2n ratio, thus triploidy could recurrently give birth to diploidy. This is namely the same as what we have found as a “nervus probandi” on Figure 4D for near-triploid endoreduplicated HeLa cells. Segregations of triploids to haploidy were also found by these authors (3n:2n:1n), but far less frequently. Although, as they also found the cytokinesis was defective or delayed, all subcells segregated after tripolar mitosis still could further divide by bipolar mitosis, except haploid YO, which existed only within a multinucleated mother. Importantly, Palitti and Rizzoni [85] scored the frequency of different variants of euploid segregations in tripolar mitoses of tetraploid and triploid cells from 77 primary diploid mouse cultures and tested as the null hypothesis if those segregate the genomes (i) in random and (ii) sending sister chromatids to different poles. This model was disproved by real scores—segregations were not random (some variants were several-fold preferred over the theoretically possible ones) and the chromatids could be not (or preferentially were not) separated, indicating a weak spindle.

Rizzoni et al [87] concluded that in mammalian somatic euploid cells there seems to exist a supra-chromosomal organization of the genome in haploid sets, which displays itself in the euploid segregations through multipolar mitosis. As to the analysis of segregation of the sex chromosomes, [86], the digynic formula 3nXXY was preferentially found, while 3n XYY (diandry) also occurred but two times less frequently. Moreover, the authors were highly surprised to reveal XX-diploid cells in male mouse fibroblast cultures after multi-polar mitoses of polyploid cells.

Finally, Pera and Scholtz [88] described the sharp conversion of the normal male mouse diploid fibroblast cell line into triploidy after ~12 months of cultivation (likely occurring due to oncogenic transformation). Two triploid cell lines comprising 85% of the population could be distinguished and isolated, one with the karyotype 3n XXY, the other with 3n XYY. In ~1% of the culture, diploid cells with two X or two Y chromosomes were found. Haploid, tetraploid, hexaploidy, and octoploid mitoses were also observed at a low percentage. The involvement of the meiotic component in tripolar segregations of diploid mammalian cells, which may be responsible for XXY karyotype and diploid XX male cells, was suggested.

We also decided to additionally extract from the Mitelman database the malignant tumor karyotypes with double X and double Y tetraploid and diploid karyotypes presented in Table 3 for comparison with the above literature data. Y0 haploid karyotypes were never encountered, and also YY were practically absent (or could exist as an exception only with fragmented X-chromosome), while XX diploid karyotypes were present in seven of 15 tumor cohorts and XXYY were present everywhere (except gastric cancer). 

Summarizing this part of the discussion, we conclude that the German and Italian groups of the 1970s revealed on normal mammalian cells the same regularity, which we were able to “fish out” using the X chromosome disomy “hook” from the 2928 male tumor karyotypes on the digyny-like origin of triploidy from Mitelman database. Without this approach, the somatic digyny in tumors is obscured by the overlaying chromosome instability and not easily seen.

Namely, these data presume the segregation of parental genomes as suprachromosomal entities, reconstruction of diploidy from triploidy and vice versa through multipolar divisions of endoreduplicated cells, as well as a preferential doubling of maternal genomes in male tumors, shown and discussed in [10].

The details of a pseudo-meiotic process remained less clear. The existence of a pseudo-meiotic process in irradiated HeLa cells was studied by Ianzini et al. [53] in collaboration with our laboratory, mostly by qPCR, where up-regulation of MOS, cyclin B1, meiotic cohesin REC8 (increased 3-fold on day three and 10-fold on day five, with repeating peaks on day 10 and 25), meiotic recombinase DMC1, and SYCP3 were found. The overexpressed meiotic protein SCP3 was even suggested as a prognostic marker for cervical carcinomas [89]. However, pseudo-meiosis in tumors seems to be occurring even without conventional synaptonemal complexes [12]. More studies on this issue are needed in the future.

Interestingly, concerning meiotic kinase MOS and quite in accord with our current data, Vitale et al [90] found in colon cancer p53-null cells treated with nocodazole that multipolar mitosis in induced transient tetraploidy cells was dependent on upregulation of this kinase, which inhibited the coalescence of supernumerary centrosomes and finally favored selection of less aneuploid progeny.

Multipolar mitosis in tumors has been extensively studied and explained by the dysfunctional spindle assembly checkpoint leading to nearly random segregation of sister chromatids between poles [91,92], then the role of cytokinesis failure in the asymmetric segregation of chromosomes into two daughter cells was highlighted [93]. However, it seems that the rules applicable to the regulation of mitosis as such may be non-applicable for tripolar mitosis in the germ-like PGCCs. Further detailed studies are needed but currently it appears that in the latter not the chromatids but the genomes are segregated, reassorted and fused in a pedogamic manner at the background of the weak spindle checkpoint. The same can be said about a-cytokinesis. In fact, radial cytokinesis releasing progeny from PGCC is postponed [12] and, by analogy with such in Drosophila egg chambers [94], is inherently unequal.

## 5. Conclusions

Considering the nature of the complex process involving the PGCC, it seems to be evolutionary pre-programmed and aimed for the atavistic response mode for cell survival in adverse conditions, operating with the whole genomes in a parasexual life cycle of tumor cells. The molecular background of the endoreduplicated pseudo-blastula in the conditions of oncogenic and/or genotoxic stress likely favors the adaptive triploid digyny-like process assisted through tripolar mitosis. The exchange between reproductive (with meiotic elements) diploid and clonogenic triploid subfractions supports the tumor immortality by recombinative genetic variation, on one side, and protects from recessive lethal mutations with the selection of the fittest clones, on the other side. These whole-genome rearrangements do not exclude chromosome instability because stochastic noise and occasional chromothripsis are also inevitably needed for the optimization of the inheritance system [95].

The digyny concept also does not contradict but essentially complement the paradigm of the somatic mutation/clonal selection origin of cancer.

## Figures and Tables

**Table 1:** Some examples of the polyclonal karyotypes (numbered in columns) from male renal carcinoma with near-triploid XXY, near-diploid, and near- tetraploid clones, extracted from the Mitelman karyotype database. (Chr—modal chromosome number, Sex—sex chromosome complement).

Case	Chr1	Sex1	Chr2	Sex2	Chr3	Sex3	Chr4	Sex4
1	70	XXY	46	XY	42	XXY	45	XXY
2	75	XXY	68	XXY	87	XXXXYY	48	XY
3	46	XX, -Y	45	XX, -Y	62	XX, -Y		
4	74	XXY	46	X, -Y	46	XY		

**Table 2:** Statistically significant and concordant increase of the proportion of diploid and tetraploid cell nuclei tested by FISH centromeres labels of three chromosomes at 48 h post 10 Gy irradiation of HeLa cells.

		Chromosome X		Chromosome 6		Chromosome 10	
Share of Cell Nuclei with a Certain Number of Centromere Signals		Number of Nuclei	%	Number of Nuclei	%	Number of Nuclei	%
Diploid and tetraploid nuclei	NT	19	3.1	17	3.1	23	3.4
Triploid and other cell nuclei	NT	586	96.9	523	96.9	649	96.6
Diploid and tetraploid nuclei	48h	53	10.2	34	6.2	54	7.0
Triploid and other cell nuclei	48h	466	89.8	518	93.8	714	93.0
χ-squared test, *p*-value		2.4·10^−20^		3.6·10^−5^		3.7·10^−12^	

**Table 3:** The proportions of tetraploid and diploid karyotypes possessing two X chromosomes, and the almost-absent karyotypes with two Y-chromosomes for 15 male malignant tumor cohorts from the Mitelman database.

Malignant Tumor Type	Number of Karyotypes	XXYY (%)	X,−Y,+X (%)	YY,−X (or with Fragmented X) (%)
Seminoma	78	12.82	2.56	0.00
Osteosarcoma	61	6.56	0	0.00
Lung carcinoma	237	2.95	0.84	0.00
Gastric carcinoma	74	0.00	4.05	0.00
Head and neck squamous cell carcinoma	191	3.14	0	0.00
Colon adenocarcinoma	98	3.06	3.06	0.00
Transitional cell carcinoma	104	0.96	0	0.00
Chondrosarcoma	85	5.88	0	0.00
Malignant melanoma	134	1.49	0.75	0.00
Glioblastoma	215	6.51	0	0.00
Renal carcinoma	577	1.39	0.87	0.17
Mesothelioma	72	5.56	0	0.00
Rhabdomyosarcoma *	92	30.43	3.26	1.09
Ewing sarcoma	228	3.51	0	0.00
Liposarcoma	147	3.40	0	0.00

* Rhabdomyosarcoma is an outlier as originating from multi-nuclear cells.

## Supplementary Materials

The following are available online at https://www.mdpi.com/2073-4425/10/7/551/s1, Figure S1: DNA content histograms of two locally advanced triple-negative breast cancer cases (Stage III) before and after neoadjuvant chemotherapy (denoted as NAC) using standard doses of paclitaxel and doxorubicin, Figure S2: Microphotograph of TB-DNA-stained diagnostic biopsy cell nuclei of the triple negative case 17.

## References

[1] Erenpreiss J. (1993). Current Concepts of Malignant Growth. Part A: From a Normal Cell to Cancer.

[2] Inhorn S.L., Therman E., Patau K. (1964). Cytogenetic studies in spontaneous human abortion. Am. J. Clin. Pathol..

[3] Hanahan D., Weinberg R.A. (2011). Hallmarks of cancer: The next generation. Cell.

[4] Sheltzer J.M., Amon A. (2011). The aneuploidy paradox: Costs and benefits of an incorrect karyotype. Trends Genet..

[5] Holland A.J., Cleveland D.W. (2012). Losing balance: The origin and impact of aneuploidy in cancer. EMBO Rep..

[6] Jo Y., Choi N., Kim K., Koo H.-J., Choi J., Kim H.N. (2018). Chemoresistance of cancer cells: Requirements of tumor microenvironment-mimicking models in anti-cancer drug development. Theranostics.

[7] Teixeira M.T., Pandis N., Heim S., Heim S., Mitelman F. (1995). Tumours of the Breast. Cancer Cytogenetics.

[8] Swanton C., Nicke B., Schuett M., Eklund A.C., Ng C., Li Q., Hardcastle T., Lee A., Roy R., East P. (2009). Chromosomal instability determines taxane response. Proc. Natl. Acad. Sci. USA.

[9] Gerashchenko B.I., Salmina K., Eglitis J., Huna A., Grjunberga V., Erenpreisa J. (2016). Disentangling the aneuploidy and senescence paradoxes: A study of triploid breast cancers non-responsive to neoadjuvant therapy. Histochem. Cell Biol..

[10] Vainshelbaum N.M., Zayakin P., Kleina R., Erenpreisa J. (2019). Meta-analysis of cancer triploidy: Whole-genome rearrangements in male human tumours are characterised by XXY karyotypes. Genes.

[11] Mitelman F., Johansson B., Mertens F. Mitelman Database of Chromosome Aberrations and Gene Fusions in Cancer. http://cgap.nci.nih.gov/Chromosomes/Mitelman.

[12] Salmina K., Huna A., Kalejs M., Pjanova D., Scherthan H., Cragg M.S., Erenpreisa J. (2019). The cancer aneuploidy paradox: In the light of evolution. Genes.

[13] Giaretti W., Venesio T., Sciutto A., Prevosto C., Geido E., Risio M. (2003). Near-diploid and near-triploid human sporadic colorectal adenocarcinomas differ for KRAS2 and TP53 mutational status. Genes Chromosomes Cancer.

[14] Kondrashov A.S. (1994). The asexual ploidy cycle and the origin of sex. Nature.

[15] Yant L., Bomblies K. (2015). Genome management and mismanagement—Cell-level opportunities and challenges of whole-genome duplication. Genes Dev..

[16] Tsang M., Gantchev J., Netchiporouk E., Moreau L., Ghazawi F.M., Glassman S., Sasseville D., Litvinov I.V. (2018). A study of meiomitosis and novel pathways of genomic instability in cutaneous T-cell lymphomas (CTCL). Oncotarget.

[17] Collins U.K. (2014). Collins English Dictionary.

[18] Stevenson A. (2010). Oxford Dictionary of English.

[19] Schinkel C.C.F., Kirchheimer B., Dullinger S., Geelen D., De Storme N., Hörandl E. (2017). Pathways to polyploidy: Indications of a female triploid bridge in the alpine species *Ranunculus kuepferi* (Ranunculaceae). Plant Syst. Evol..

[20] Comai L. (2005). The advantages and disadvantages of being polyploid. Nat. Rev. Genet..

[21] Sundaram M., Guernsey D.L., Rajaraman M.M., Rajaraman R. (2004). Neosis: A novel type of cell division in cancer. Cancer Biol. Ther..

[22] Rajaraman R., Rajaraman M.M., Rajaraman S.R., Guernsey D.L. (2005). Neosis—A paradigm of self-renewal in cancer. Cell Biol. Int..

[23] Harlan J.R., deWet J.M.J., On Ö. (1975). Winge and a Prayer: The origins of polyploidy. Bot. Rev..

[24] Otto S.P. (2007). The evolutionary consequences of polyploidy. Cell.

[25] Pawlowitzki I.H., Cenani A. (1967). Sporadic triploid cells in human blood and fibroblast cultures. Humangenetik.

[26] Austin C.R. (1960). Anomalies of fertilization leading to triploidy. J. Cell. Comp. Physiol..

[27] Neaves W.B., Baumann P. (2011). Unisexual reproduction among vertebrates. Trends Genet..

[28] Daniel A., Wu Z., Darmanian A., Collins F., Jackson J. (2003). Three different origins for apparent triploid/diploid mosaics. Prenat. Diagn..

[29] Mason A.S., Pires J.C. (2015). Unreduced gametes: Meiotic mishap or evolutionary mechanism?. Trends Genet..

[30] Roeder G.S. (1997). Meiotic chromosomes: It takes two to tango. Genes Dev..

[31] Zhou L., Gui J. (2017). Natural and artificial polyploids in aquaculture. Aquac. Fish..

[32] Sell S., Nicolini A., Ferrari P., Biava P.M. (2016). Cancer: A problem of developmental biology; scientific evidence for reprogramming and differentiation therapy. Curr. Drug Targets.

[33] Yeom Y.I., Fuhrmann G., Ovitt C.E., Brehm A., Ohbo K., Gross M., Hübner K., Schöler H.R. (1996). Germline regulatory element of Oct-4 specific for the totipotent cycle of embryonal cells. Development.

[34] Salmina K., Jankevics E., Huna A., Perminov D., Radovica I., Klymenko T., Ivanov A., Jascenko E., Scherthan H., Cragg M. (2010). Up-regulation of the embryonic self-renewal network through reversible polyploidy in irradiated p53-mutant tumour cells. Exp. Cell Res..

[35] Erenpreisa J., Salmina K., Huna A., Jackson T.R., Vazquez-Martin A., Cragg M.S. (2015). The “virgin birth”, polyploidy, and the origin of cancer. Oncoscience.

[36] Niu N., Mercado-Uribe I., Liu J. (2017). Dedifferentiation into blastomere-like cancer stem cells via formation of polyploid giant cancer cells. Oncogene.

[37] Ben-Porath I., Thomson M.W., Carey V.J., Ge R., Bell G.W., Regev A., Weinberg R.A. (2008). An embryonic stem cell-like gene expression signature in poorly differentiated aggressive human tumors. Nat. Genet..

[38] Shaffer S.M., Dunagin M.C., Torborg S.R., Torre E.A., Emert B., Krepler C., Beqiri M., Sproesser K., Brafford P.A., Xiao M. (2017). Rare cell variability and drug-induced reprogramming as a mode of cancer drug resistance. Nature.

[39] Kim C., Gao R., Sei E., Brandt R., Hartman J., Hatschek T., Crosetto N., Foukakis T., Navin N.E. (2018). Chemoresistance evolution in triple-negative breast cancer delineated by single-cell sequencing. Cell.

[40] Cohnheim J. (1880). Vorlesungen uber Allgemeine Pathologie. Ein Handbuch fur Arzte und Studierende.

[41] Barry Pierce G., Johnson L.D. (1971). Differentiation and cancer. In Vitro.

[42] Pierce G.B. (1983). The cancer cell and its control by the embryo. Rous-Whipple Award lecture. Am. J. Pathol..

[43] Virchow R., Chance F., Goodsir J., Osborn S., King’s College London, Pathological Institute of Berlin, St. Thomas’s Hospital (1860). Cellular Pathology as Based upon Physiological and Pathological Histology.

[44] Ērenpreisa J., Dālmane A., Ērenpreiss J. (2000). Jānis Oļģerts Ērenpreiss and his theory of carcinogenesis. Acta Med. Hist. Rigensia Riga.

[45] Vinnitsky V. (2014). The development of a malignant tumor is due to a desperate asexual self-cloning process in which cancer stem cells develop the ability to mimic the genetic program of germline cells. Intrinsically Disord. Proteins.

[46] Cofre J., Abdelhay E. (2017). Cancer is to embryology as mutation is to genetics: Hypothesis of the cancer as embryological phenomenon. Sci. World J..

[47] Chen J., Niu N., Zhang J., Qi L., Shen W., Donkena K.V., Feng Z., Liu J. (2019). Polyploid giant cancer cells (PGCCs): The evil roots of cancer. Curr. Cancer Drug Targets.

[48] Erenpreisa J., Cragg M.S. (2007). Cancer: A matter of life cycle?. Cell Biol. Int..

[49] Buiķis I., Harju L., Freivalds T. (1999). Origin of microcells in the human sarcoma cell line HT-1080. Anal. Cell. Pathol..

[50] Zhang S., Mercado-Uribe I., Xing Z., Sun B., Kuang J., Liu J. (2014). Generation of cancer stem-like cells through the formation of polyploid giant cancer cells. Oncogene.

[51] Kalejs M., Ivanov A., Plakhins G., Cragg M.S., Emzinsh D., Illidge T.M., Erenpreisa J. (2006). Upregulation of meiosis-specific genes in lymphoma cell lines following genotoxic insult and induction of mitotic catastrophe. BMC Cancer.

[52] Erenpreisa J., Cragg M.S., Salmina K., Hausmann M., Scherthan H. (2009). The role of meiotic cohesin REC8 in chromosome segregation in γ irradiation-induced endopolyploid tumour cells. Exp. Cell Res..

[53] Ianzini F., Kosmacek E.A., Nelson E.S., Napoli E., Erenpreisa J., Kalejs M., Mackey M.A. (2009). Activation of meiosis-specific genes is associated with depolyploidization of human tumor cells following radiation-induced mitotic catastrophe. Cancer Res..

[54] Erenpreisa J., Kalejs M., Cragg M.S. (2005). Mitotic catastrophe and endomitosis in tumour cells: An evolutionary key to a molecular solution. Cell Biol. Int..

[55] Gorgoulis V.G., Zacharatos P., Mariatos G., Liloglou T., Kokotas S., Kastrinakis N., Kotsinas A., Athanasiou A., Foukas P., Zoumpourlis V. (2001). Deregulated expression of c-*mos* in non-small cell lung carcinomas: Relationship with *p53* status, genomic instability, and tumor kinetics. Cancer Res..

[56] Klymiuk I., Kenner L., Adler T., Busch D.H., Boersma A., Irmler M., Fridrich B., Gailus-Durner V., Fuchs H., Leitner N. (2012). In vivo functional requirement of the mouse *Ifitm1* gene for germ cell development, interferon mediated immune response and somitogenesis. PLoS ONE.

[57] Grell K.G. (1953). Die Chromosomen von Aulacantha scolymantha Haeckel. Arch. Protistenkd.

[58] Raĭkov I.B. (1982). The Protozoan Nucleus, Morphology and Evolution.

[59] Huskins C.L. (1948). Chromosome multiplication and reduction in somatic tissues; their possible relation to differentiation, reversion and sex. Nature.

[60] Walen K. (2013). Normal human cells acquiring proliferative advantage to hyperplasia-like growth-morphology: Aberrant progeny cells associated with endopolyploid and haploid divisions. Cancer Clin. Oncol..

[61] Walen K.H. (2014). Neoplastic-like cell changes of normal fibroblast cells associated with evolutionary conserved maternal and paternal genomic autonomous behavior (Gonomery). J. Cancer Ther..

[62] Delhanty J.D., Handyside A.H. (1995). The origin of genetic defects in the human and their detection in the preimplantation embryo. Hum. Reprod. Update.

[63] Zernicka-Goetz M., Huang S. (2010). Stochasticity versus determinism in development: A false dichotomy?. Nat. Rev. Genet..

[64] Vanneste E., Voet T., Le Caignec C., Ampe M., Konings P., Melotte C., Debrock S., Amyere M., Vikkula M., Schuit F. (2009). Chromosome instability is common in human cleavage-stage embryos. Nat. Med..

[65] Hansis C. (2001). Analysis of Oct-4 expression and ploidy in individual human blastomeres. Mol. Hum. Reprod..

[66] Wolpert L., Beddington R., Jessell T., Lawrence P., Meyerowitz E., Smith J. (2002). Principles of Development.

[67] Mayer W., Smith A., Fundele R., Haaf T. (2000). Spatial separation of parental genomes in preimplantation mouse embryos. J. Cell Biol..

[68] Illidge T., Cragg M., Fringe B., Olive P., Erenpreisa J. (2000). Polyploid giant cells provide a survival mechanism for p53 mutant cells after DNA damage. Cell Biol. Int..

[69] Erenpreisa J.A., Cragg M.S., Fringes B., Sharakhov I., Illidge T.M. (2000). Release of mitotic descendants by giant cells from irradiated Burkitt’s lymphoma cell line. Cell Biol. Int..

[70] Erenpreisa J., Kalejs M., Ianzini F., Kosmacek E.A., Mackey M.A., Emzinsh D., Cragg M.S., Ivanov A., Illidge T.M. (2005). Segregation of genomes in polyploid tumour cells following mitotic catastrophe. Cell Biol. Int..

[71] Erenpreisa J., Cragg M.S., Anisimov A.P., Illidge T.M. (2011). Tumor cell embryonality and the ploidy number 32n: Is it a developmental checkpoint?. Cell Cycle.

[72] Weihua Z., Lin Q., Ramoth A.J., Fan D., Fidler I.J. (2011). Formation of solid tumors by a single multinucleated cancer cell. Cancer.

[73] Mirzayans R., Andrais B., Murray D. (2018). Roles of polyploid/multinucleated giant cancer cells in metastasis and disease relapse following anticancer treatment. Cancers.

[74] Rosenbusch B.E. (2008). Mechanisms giving rise to triploid zygotes during assisted reproduction. Fertil. Steril..

[75] Kalatova B., Jesenska R., Hlinka D., Dudas M. (2015). Tripolar mitosis in human cells and embryos: Occurrence, pathophysiology and medical implications. Acta Histochem..

[76] Schwarz-Finsterle J., Scherthan H., Huna A., González P., Mueller P., Schmitt E., Erenpreisa J., Hausmann M. (2013). Volume increase and spatial shifts of chromosome territories in nuclei of radiation-induced polyploidizing tumour cells. Mutat. Res..

[77] Macville M., Schröck E., Padilla-Nash H., Keck C., Ghadimi B.M., Zimonjic D., Popescu N., Ried T. (1999). Comprehensive and definitive molecular cytogenetic characterization of HeLa cells by spectral karyotyping. Cancer Res..

[78] Erenpreisa J., Ivanov A., Wheatley S.P., Kosmacek E.A., Ianzini F., Anisimov A.P., Mackey M., Davis P.J., Plakhins G., Illidge T.M. (2008). Endopolyploidy in irradiated p53-deficient tumour cell lines: Persistence of cell division activity in giant cells expressing Aurora-B kinase. Cell Biol. Int..

[79] Ghosh S., Ghosh I. (1975). Variation of stemline karyotype in a HeLa cell line. Z. Krebsforsch. Klin. Onkol..

[80] Ianzini F., Mackey M.A. (2002). Development of the large scale digital cell analysis system. Radiat. Prot. Dosim..

[81] Erenpreisa J., Salmina K., Huna A., Kosmacek E.A., Cragg M.S., Ianzini F., Anisimov A.P. (2011). Polyploid tumour cells elicit paradiploid progeny through depolyploidizing divisions and regulated autophagic degradation. Cell Biol. Int..

[82] Kröger C., Afeyan A., Mraz J., Eaton E.N., Reinhardt F., Khodor Y.L., Thiru P., Bierie B., Ye X., Burge C.B. (2019). Acquisition of a hybrid E/M state is essential for tumorigenicity of basal breast cancer cells. Proc. Natl. Acad. Sci. USA.

[83] Navin N.E. (2015). The first five years of single-cell cancer genomics and beyond. Genome Res..

[84] Pera F., Schwarzacher H.G. (1969). Die Verteilung der Chromosomen auf die Tochterzellkerne multipolarer Mitosen in euploiden Gewebekulturen von Microtus agrestis. Chromosoma.

[85] Palitti F., Rizzoni M. (1972). Pattern of DNA segregation in multipolar anatelophases of different ploidy in euploid and aneuploid mammalian cells cultivated in vitro. Genetica.

[86] Pera F., Rainer B. (1973). Studies of multipolar mitoses in euploid tissue cultures. I. Somatic reduction to exactly haploid and triploid chromosome sets. Chromosoma (Berl.).

[87] Rizzoni M., Palitti F., Perticone P. (1974). Euploid segregation through multipolar mitosis in mammalian cell cultures. Identification of triploid, haploid, and segregating diploid cells in a diploid-euploid primary culture of rhesus kidney cells. Chromosoma.

[88] Pera F., Scholz P. (1974). Polyploidization in vitro: Formation of a predominantly triploid cell population in an originally diploid tissue culture of *Microtus agrestis*. Hum. Genet..

[89] Cho H., Noh K.H., Chung J.-Y., Takikita M., Chung E.J., Kim B.W., Hewitt S.M., Kim T.W., Kim J.-H. (2014). Synaptonemal complex protein 3 is a prognostic marker in cervical cancer. PLoS ONE.

[90] Vitale I., Senovilla L., Jemaà M., Michaud M., Galluzzi L., Kepp O., Nanty L., Criollo A., Rello-Varona S., Manic G. (2010). Multipolar mitosis of tetraploid cells: Inhibition by p53 and dependency on Mos. EMBO J..

[91] Stewenius Y., Gorunova L., Jonson T., Larsson N., Hoglund M., Mandahl N., Mertens F., Mitelman F., Gisselsson D. (2005). Structural and numerical chromosome changes in colon cancer develop through telomere-mediated anaphase bridges, not through mitotic multipolarity. Proc. Natl. Acad. Sci. USA.

[92] Gisselsson D., Håkanson U., Stoller P., Marti D., Jin Y., Rosengren A.H., Stewénius Y., Kahl F., Panagopoulos I. (2008). When the genome plays dice: Circumvention of the spindle assembly checkpoint and near-random chromosome segregation in multipolar cancer cell mitoses. PLoS ONE.

[93] Gisselsson D., Jin Y., Lindgren D., Persson J., Gisselsson L., Hanks S., Sehic D., Mengelbier L.H., Øra I., Rahman N. (2010). Generation of trisomies in cancer cells by multipolar mitosis and incomplete cytokinesis. Proc. Natl. Acad. Sci. USA.

[94] Lin H., Yue L., Spradling A.C. (1994). The Drosophila fusome, a germline-specific organelle, contains membrane skeletal proteins and functions in cyst formation. Development.

[95] Ye C.J., Regan S., Liu G., Alemara S., Heng H.H. (2018). Understanding aneuploidy in cancer through the lens of system inheritance, fuzzy inheritance and emergence of new genome systems. Mol. Cytogenet..

