# Supplementary material for: When Three Isn’t a Crowd: A Digyny Concept for Treatment-Resistant, Near-Triploid Human Cancers
Source: Genes (Basel). 2019 Jul 19;10(7):551. doi: 10.3390/genes10070551 (PMC6678365; doi:10.3390/genes10070551)
Supplement: Supplementary file 1 [file genes-10-00551-s001.zip › Suppl Fig.S2.pdf]

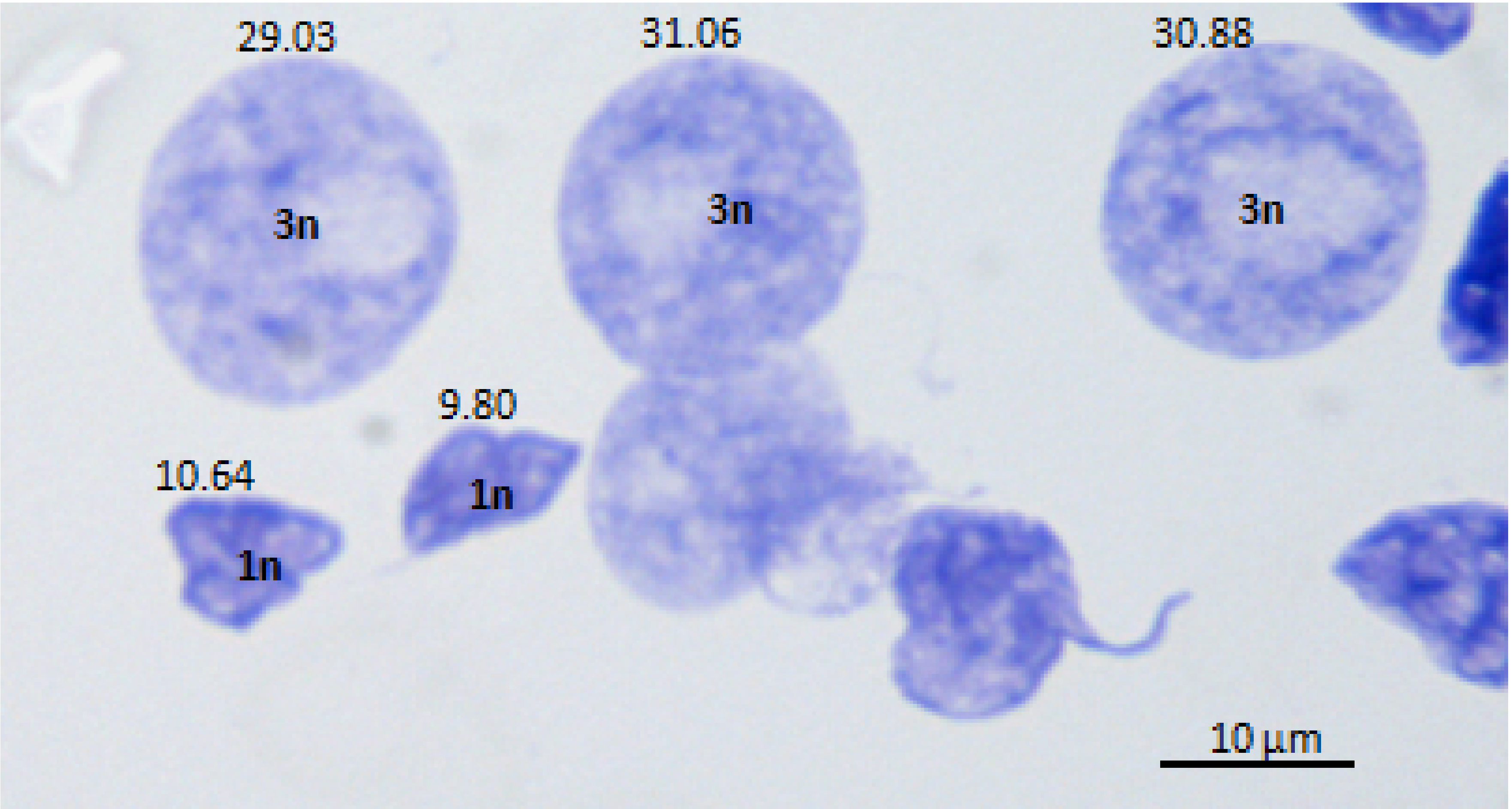

Supplementary Fig.S2. Microphotograph of TB-DNA-stained diagnostic biopsy cell nuclei of the triple negative case 17. Among triploid nuclei (3n), a pair of haploid nuclei (1n) is seen, whose morphology and a faint bridge indicate that they have derived by divison of a common mother . The numeric values shown next to the nuclei represent IODs of these nuclei. Average IOD for lymphocytes (2c) = 19.93 ± 1.39 SD (n = 5).
