# Supplementary material for: When Three Isn’t a Crowd: A Digyny Concept for Treatment-Resistant, Near-Triploid Human Cancers
Source: Genes (Basel). 2019 Jul 19;10(7):551. doi: 10.3390/genes10070551 (PMC6678365; doi:10.3390/genes10070551)
Supplement: Supplementary file 1 [file genes-10-00551-s001.zip › Suppl. Fig.S1.pdf]

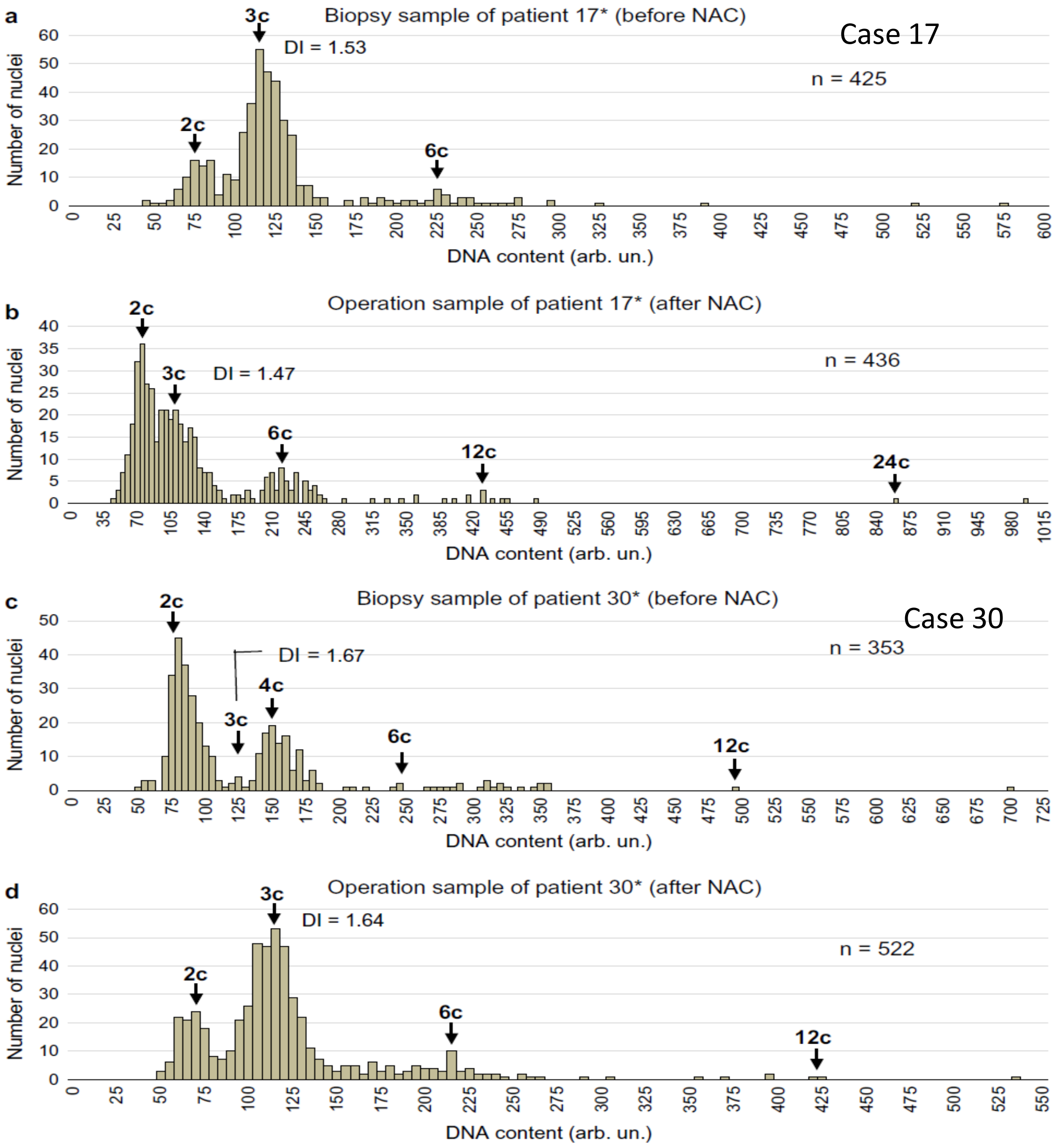

Suppl. Fig. S1. DNA content histograms of two locally advanced triple-negative breast cancer cases (Stage III) before and after neoadjuvant chemotherapy (denoted as NAC) using standard doses of paclitaxel and doxorubicin. DNA index (DI) shows the ratio of the DNA content in G1 of the given stemline to the G1/DNA content of the diploid stemline.In case 17 (a, b), the triploid (3C) stemline, which was dominant in biopsy before NAC (a), was still present in operation material after NAC accompanied by increased fractions of corresponding 6C, 12C and 24C cells. (b). According to Miller-Payne classification [Ogston KN, Miller ID, Payne S, et al. A new histological grading system to assess response of breast cancers to primary chemotherapy: prognostic significance and survival. *Breast* 2003; 12(5): 320–327.], this case belongs to the Grade 1 (non-responsive cancer). As for case 30 (c, d), biopsy (c) showed the nearly normal histogram with a very small fraction of ~3C cells (DI = 1.67). whose presence can be confirmed by appearance corresponding duplicates (6C) and quadruplicates (12C). However, as a result of NAC (d), this minor proliferating stemline suddenly became dominant accompanied by an increase of fractions of 6C and 12C cells. According to Miller-Payne classification, this case belongs to the Grades 1–2 (non-responsive cancer). Republished from Gerashchenko with permission [9].
